# Antipostmenopausal effects of *Stauntonia hexaphylla* and *Vaccinium bracteatum* fruit combination in estrogen-deficient rats

**DOI:** 10.29219/fnr.v64.5233

**Published:** 2020-10-15

**Authors:** Gyuok Lee, Jawon Shin, Ara Jo, Sojeong Lm, Mi-Ri Kim, Yunhee Shoi, Hyojeong Yun, Donghyuck Bae, Jaeyong Kim, Chul-yung Choi

**Affiliations:** Jeonnam Bioindustry Foundation, Jeonnam Institute of Natural Resources Research (JINR), Jeollanamdo, Republic of Korea

**Keywords:** estrogen, hot flushes, menopause, osteoporosis, ovariectomy

## Abstract

**Background:**

Climacterium is a series of physical and mental symptoms occurring in women and men due to decreased levels of sex hormones. Women lose the ability to become pregnant due to decreased ovarian estrogen production; the initial symptom being hot flushes. In addition, urogenital atrophy, sexual dysfunction, mood changes, and osteoporosis occur. Extracts of *Stauntonia hexaphylla* (SH) and *Vaccinium bracteatum* (VB) fruits, with a wide range of biological activities, are widely used in traditional herbal medicine.

**Objective:**

The purpose of this study was to investigate the mitigation of menopausal symptoms, such as hot flushes and postmenopausal osteoporosis after combinatorial treatment with SH and VB (SHVB) of ovariectomized (OVX) rats.

**Design:**

We measured the bone regenerative effect of SHVB on receptor activator of nuclear factor-κB (NF-κB) ligand-induced osteoclast differentiation *in vitro* and on ovariectomy-induced osteoporosis *in vivo*. We investigated the effect of SHVB in a rat model of menopausal hot flushes, in which the tail skin temperature increases following ovariectomy-induced rapid decline in estrogen levels.

**Results:**

SHVB inhibited osteoclast formation and tartrate-resistant acid phosphatase activity in primary mouse bone marrow-derived cells. In an estrogen deficiency-induced rat model, measurement of serum bone turnover factors showed that treatment with SHVB lowered the increased bone turnover. Additionally, SHVB decreased OVX-induced bone loss of the total femur. SHVB inhibited osteoclast differentiation, prevented bone mass reduction, and improved trabecular bone structure and biochemical markers in OVX-induced osteoporosis. In addition, administration of SHVB significantly ameliorated the changes in skin temperature in OVX rats.

**Conclusion:**

SHVB improved the symptoms of menopause. These results provide the foundation for developing SHVB as a natural substance to replace hormones in the future.

## Popular scientific summary

- *Stauntonia hexaphylla* and *Vaccinium bracteatum* (SHVB) inhibited the elevation of tail skin temperature potentiation without affecting the weight of the uterus and serum estradiol in ovariectomized rats.
- SHVB ameliorated osteoporotic parameters in serum and prevented trabecular microarchitecture deterioration.

Menopause is the final menstrual period in the aging process and marks the permanent cessation of menstrual cycles following the loss of ovarian follicular activity (1). As ovarian hormones, such as estrogen, decrease rapidly before and after menopause, various symptoms including physiological, physical, and mental changes, collectively called the climacterium syndrome, are known to accompany menopause (2). Menopausal women commonly experience symptoms including osteoporosis, hot flushes, hyperlipidemia, and cardiovascular disease, which have been associated with the decrease in the endogenous levels of estrogen (3).

Osteoporosis is known to be an age-dependent multifunctional skeletal disease characterized by decreased bone mineral density (BMD), deterioration of bone microarchitecture, and increased risk of fragility fractures (4, 5). Physiologically, bone mass is known to be balanced through bone remodeling processes involving bone-forming osteoblasts and bone-resorbing osteoclasts (6, 7). Especially, osteoclasts, which are bone-resorbing multinucleated giant cells, are differentiated from hemopoietic progenitors of monocyte–macrophage lineage precursor cells following their activation by crucial cytokines: macrophage colony-stimulating factor (M-CSF) and receptor activator of nuclear factor-κB (NF-κB) ligand (RANKL) (8). In particular, M-CSF has been shown to play a crucial role in the proliferation and survival of osteoclast precursors and the constitutive expression of RANK (9).

In preclinical and clinical research, bone turnover biomarkers are typically categorized into bone formation and resorption markers (10). Accordingly, bone formation markers are osteoblastic enzymes or by-products of active osteoblasts produced during the various phases of bone development. In contrast, most bone resorption markers are degradation products, such as collagen type I, non-collagenous bone matrix proteins, and osteoclastic enzymes. In addition, several regulators of the activity of bone cells and, therefore, of bone turnover might also be used as biomarkers. Bone turnover biomarkers used in preclinical and clinical research of bone metastasis usually include serum bone-specific alkaline phosphatase (BALP), osteocalcin (OCN), and procollagen type I N propeptide, as well as pyridinoline, deoxypyridinoline, aminoterminal crosslinked telopeptide of type I collagen (NTX-I), and carboxy-terminal crosslinked telopeptides of collagen type I (CTx-I and ICTP) (11).

Menopause transition refers to the time from the onset of changes in the menstrual cycle or vasomotor symptoms until a year following the final menstrual period (12). Most women entering menopause have been reported to experience vasomotor symptoms. A hot flush is a sudden episode of vasodilation in the face and neck, lasting 1–5 min, and accompanied by profuse sweating. Women who experience hot flushes are reported to have a narrower thermoneutral zone, such that subtle changes in the core temperature might elicit thermoregulatory mechanisms, such as vasodilation, sweating, or shivering (13). The aspects of the physiology of hot flushes on temperature regulation are not known in detail, but probably involve the core body temperature; central processing areas in the CNS, neuromodulators, peripheral vasculature, and sweat glands (14). Hot flushes have been shown to occur as a transient increase in the temperature of the skin, associated with objective signs of cutaneous vasodilation and vasoconstriction when the core temperature drops (15).

Estrogen-based hormonal therapy has been used as an effective treatment for hot flushes. However, results obtained from numerous clinical trials indicated increased problems of thromboembolic incidents, heart disease, and breast cancer in women receiving long-term hormone treatment therapy (16). Therefore, as an alternative to hormone replacement therapy (HRT), selective serotonin reuptake inhibitors and venlafaxine have been introduced as a primary therapy for hot flushes (17).

The occurrence of osteoporosis is known to increase with age, and most frequently in postmenopausal women owing to the menopausal decline in the levels of ovarian hormones (18). In addition, estrogen deficiency has been demonstrated to impair the trabecular metaphyseal bone, reducing the bone mass in humans and animals (19, 20). HRT has proven efficacious in preventing osteoporosis and reducing the incidence of bone fractures in postmenopausal women (21). However, long-term HRT has been shown to be accompanied by estrogen-like side effects, such as breast and endometrial cancers (22). Therefore, non-hormonal or herbal medicine therapies might be safer and, therefore, more acceptable alternative options for the treatment and prevention of osteoporosis without any adverse effects.

The *Stauntonia hexaphylla* (SH) plant belongs to *Lardizabalaceae* and is widely distributed in Korea, Japan, and China. It has been commonly used as a nutrient-dense food in China, especially for its analgesic, sedative, and diuretic properties (23–25). Previous studies have demonstrated that the leaves of SH exerted pharmacological effects, including antidiabetic (26) and antiosteoporosis (27) effects. We have also previously reported that the fruit extract of SH exhibited antiinflammatory effects in lipopolysaccharide-activated RAW 264.7 cells and in carrageenan-induced paw edema rats (28). We also reported that the fruit extract of SH demonstrated antioxidant and hepatoprotective effects on hydrogen peroxide-induced cytotoxicity in HepG2 cells (29). The *Vaccinium bracteatum* (VB) plant belongs to the *Ericaceae* genus, and its fruits are commonly known in Korea as the ‘oriental blueberry’. Previous studies have demonstrated that VB exerted various pharmacological effects, including antifatigue (30), antimicrobial (31), antidiabetic (32, 33), antioxidant (34), retinal protection (35), antiproliferative, and antiinflammatory effects (36, 37). We have previously reported that the fruit of VB exhibited antidepressant properties, protective effects on oxidative stress-induced apoptosis, sedative, and hypnotic effects (38–40). In addition, we have also reported the antidepressant-like effects of the combination of fruit extracts of SH and VB (SHVB), especially in models of congenital rubella syndrome and related depression disorders (41). There have been no studies showing whether the combination of fruit extracts of SHVB might be effective in treating hot flushes and osteoporosis, which are both symptoms of female menopause. In the present study, we evaluated the effects of SHVB on preventing hot flushes, osteoporosis, and ameliorating bone loss in ovariectomized (OVX) rats.

## Materials and methods

### Preparation of plant extract

Fruits of SH (Thunb.) Decne. were collected from plants in the Jangheung-gun County (Jeollanamdo, Republic of Korea). Fruits of VB Thunb. used in this study were collected from plants in the Goheung County (Jeollanamdo, Republic of Korea). The active ingredients were extracted with water at 100°C for 4 h. Accordingly, the extraction yield of the fruits of SH (Thunb.) Decne. and VB Thunb. was about 8.0 and 12.5%, respectively. Extracts were stored at 4°C until further use. The SHVB (NET-1601, SH:VB = 1:1, w/w) sample used in the present study was also used in the clinical trial, which was approved by the Institutional Review Board at Konkuk University Medical Center (clinical trials registration number KUMC 2019-07-033-001).

### High-performance liquid chromatography analysis

Analysis of the obtained extracts was performed using the SHIMADZU series ultra-fast liquid chromatography system (LC-20AD, Shimadzu, Kyoto, Japan), which comprised a diode array detector (SPD-M20A). The column used was a Carotenoid-C30 (250 × 4.6 mm, 5 μm, YMC, Japan), and the detection wavelength was set at 340 nm for the combined extract of SHVB. The temperature of the column was set to 35°C. Mobile phase A was water, while mobile phase B was acetonitrile with the elution profile being as follows: 86–84% A, 0–40 min, 84–100% A; 40–42 min, 100% A; 42–52 min, 100–86% A; 52–53 min, 86% A; 53–60 min. The flow rate was 1 mL/min, and the injection volume was 10 μL. These analyses were approved by the Korea Health Supplement Institute (approval no. D2019072484).

### Experiment 1: measurement of osteoporosis

#### Animals

Female Sprague–Dawley rats (230–250 g, 12 weeks old) were purchased from RNSkorea (Cheong-ju, Chungcheongbuk-do, Korea). Animals were acclimated to standard laboratory conditions with free access to food and water for 1 week before the experiment. The temperature was thermostatically regulated to 23 ± 2°C, and a 12-h light–dark schedule was maintained. Prior to the experimental procedures, rats were allowed a 1-week acclimatization to the experimental environment. All animal experimental protocols were approved by the Animal Ethical Committee of Jeollanamdo Institute for Natural Resources Research (JINR1908-2019). All experimental procedures were undertaken in compliance with the Guide for the Care and Use of Laboratory Animals (National Institutes of Health, Bethesda, MD, USA) and the National Animal Welfare Law of the Republic of Korea. At the 13th week, 30 and 10 Sprague – Dawley rats were bilaterally OVX and sham operated (Sham), respectively. After the 1-week recovery following the surgery, the OVX rats were randomly divided into three groups of 10 rats each and treated as indicated: OVX-administered vehicle (OVX); OVX-administered 91 μg/kg of 17β-estradiol (Sigma-Aldrich, St. Louis, MO, USA; E2); and OVX-administered 100 mg/kg of SHVB (SHVB). The experimental dose for E2 in the present study was equivalent to the corresponding clinical prescription dose for a 60 kg human subject. OVX, E2, and SHVB wereorally administered by mixing with distilled water (0.3 mL) for 12 weeks. After 12 weeks of treatment, animals were euthanized, and blood samples were collected for serum isolation. The femur bones were dissected, and the soft tissue removed to enable the analysis of the trabecular microarchitecture.

#### Measurements of osteocalcin, bone alkaline phosphatase, tartrate-resistant acid phosphatase, and C-terminal telopeptide in serum

The serum concentrations of OCN and the activity of BALP were assayed using a Rat Gla-Osteocalcin EIA kit (Takara Bio Inc., Otsu, Japan) and BALP assay kit (Bioassay Technology Laboratory, Shanghai, China), respectively, according to the manufacturers’ instructions. The serum levels of estradiol (E2) were also analyzed using commercial ELISA kits (ENZO, Farmingdale, NY, USA). The concentration of tartrate-resistant acid phosphatase (TRAP) was determined using a rat TRAP assay kit (Takara Bio Inc., Tokyo, Japan).

#### Bone structure analysis

Microcomputed tomography (micro-CT; Skyscan 1176, Bruker-microCT, Kontich, Belgium) was performed on distal right femurs. The X-ray source was set at a voltage of 65 kV and a current of 385 μA, and filtered with a 1 mm aluminum filter. The resolution was set at 16.93 μm and the rotation step at 0.3°. Accordingly, both 2D and 3D images were obtained for visualization and display. The structural parameters for the trabecular bone were analyzed using the CTAn software (CT analyzer V 1.13, Skyscan, Bruker-microCT, Kontich, Belgium). The structural parameters for the trabecular bone were derived from micro-CT data, including trabecular separation (Tb.Sp; mm), trabecular number (Tb.N; mm^−1^), bone volume/tissue volume (BV/TV; %), trabecular thickness (Tb.Th; mm), and structure model index (SMI), which were evaluated on the basis of traditional static bone histomorphometry.

#### Preparation of histological specimens

The excised left femurs were fixed in 4% paraformaldehyde for 24 h, and then decalcified in 10% Ethylenediaminetetraacetic acid at 4°C for 4 weeks. Then, the decalcified samples were dehydrated in an ethanol gradient of 80, 90, and 100% for 2 days at each step, defatted in xylene for 2 days, and embedded in plastic polymer. Then, the decalcified sections (5 μm) were cut using a microtome (Reichert-Jung 2040, Leica, Heidelberger, Germany) and stained with hematoxylin and eosin (H&E).

#### Cell cultures

Bone marrow (BM) cells were isolated from 12-week-old mice from the Institute of Cancer Research (ICR). In brief, BM from the tibia and femur of each mouse was flushed with Hank’s balanced salt solution, and cells were seeded in 24-well plates in 30 ng/mL M-CSF for 24 h. Next, cells were treated with the indicated concentrations of SHVB and E2 added in the osteoclastogenic medium (30 and 100 ng/mL M-CSF and RANKL, respectively) for 5 days. The medium was replaced with a 50% volume with and without SHVB every 2 days. E2 was purchased from Sigma-Aldrich (St Louis, MO, USA). Recombinant soluble human M-CSF and RANKL were obtained from Peprotech (Rocky Hill, NJ, USA). All the samples were suspended in dimethyl sulfoxide.

#### Tartrate-resistant acid phosphatase staining

Osteoclast differentiation was assessed by analyzing the activity of TRAP. Briefly, 5 days after stimulating the cells with M-CSF and RANKL (30 and 100 ng/mL, respectively), the cell culture supernatant was collected. To measure the activity of TRAP, the reaction mixture was transferred to new plates containing an equal volume of 0.1 M sodium hydroxide (NaOH), and the absorbance was measured using a microplate reader at 410 nm. The measured activity of TRAP was expressed as a percentage (%) of the control.

### Experiment 2: measurement of hot flushes

#### Animals

This study was approved by the Animal Ethical Committee of Jeollanamdo Institute for Natural Resources Research (JINR-1816-2018). Briefly, 10-week-old female Sprague–Dawley rats weighing 210–230 g were purchased from Samtako (Osan-si, Gyeonggi-do, Korea). Animals were allowed free access to tap water and standard laboratory food *ad libitum* and were housed in polycarbonate cages at a temperature of 23 ± 2°C, relative humidity of 55 ± 10%, and a 12:12 h light:dark cycle, with lights on from 07:00 to 19:00 h daily. Rats were randomly allocated into two groups before the operation. Both groups were anesthetized with intraperitoneal injection of 50 mg/kg Zoletil 50 (Virbac, Nice, France). Then, one group received bilateral ovariectomy using the dorsal approach (OVX; *n* = 30), while the other group underwent a sham operation (Sham; *n* = 10), as control. The OVX rats were then randomly divided into three groups: OVX group (OVX), E2 treatment group (E2), and SHVB treatment group (SHVB). Accordingly, SHVB (100 mg/kg body weight/day), E2 (91 μg/kg body weight/day), or distilled water as vehicle were orally administered to rats at 0.05 mL/1.0 kg body weight once a day for 4 weeks, starting 1 week after surgery. Distilled water (10 mg/kg) used as control was administered to the sham-operated rats for 4 weeks following the same schedule. On the day of measurement, OVX, E2, and SHVB were administered orally 30 min before the measurement of tail skin temperature (TST).

The uterus index (mg/g) was calculated by dividing the uterus by the body weight.

#### Measurement and analyses of tail skin temperature in rats

Rats were restrained in a holder in a conscious state, and their TST at the dorsal surface of the tail about 2 cm from the fur line was measured for 15 min using an infrared camera (Flir T650sc, FLIR Systems Inc., Portland, USA). Before testing, all animals were settled in the laboratory room for 15 min at an environmental temperature of 25°C. In the following image analysis, the highest temperature was recorded after designating the region of interest at the dorsal surface of the tail about 2 cm from the fur line of the rat using the ResearchIR v4 program. Respectively, TST data were measured at 1-min intervals throughout the experimental period. The mean TST during the 15 min of the measurement period was calculated, and the data were analyzed as the change in the mean TST for each 15 min measurement compared with the mean TST at 0 min. Changes in TST were assessed using ΔTST.

ΔTST= (tail skin temperature in each 15-min block) − (tail skin temperature at 0 min)

Values were expressed as means ± standard error (S.E.)

#### Measurements of estradiol in serum and uterus index

The serum levels of estradiol (E2) were also analyzed using commercial ELISA kits (ENZO), according to the manufacturers’ instructions.

The uterus index (mg/g) was calculated by dividing the uterus by the body weight.

#### Statistical analysis

Results were expressed as mean ± standard error of the mean (S.E.), and group data were compared using an analysis of variance (ANOVA), followed by Dunnet’s *post hoc* test. All statistical analyses were performed using the GraphPad Prism 5 for Windows (GraphPad Software, San Diego, CA, USA). A difference was considered statistically significant at *P*-value < 0.05.

## Results

### SHVB inhibited RANKL-induced osteoclastogenesis in vitro

**Fig. 1:**
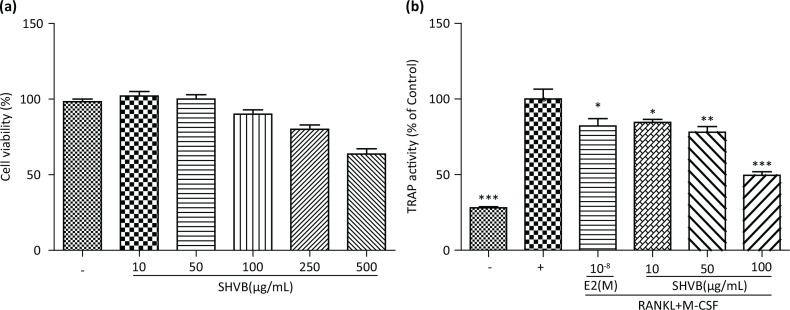
SHVB inhibits RANKL-induced osteoclastogenesis *in vitro*. (a) Cell viability was measured using the MTT method. (b) Bone marrow cells (1 × 10^4^ cells/mL) were incubated with SHVB in the presence of M-CSF (30 ng/mL) and RANKL (100 ng/mL) for 5 days. Osteoclastogenesis was confirmed by TRAP staining. Data represent the mean ± S.E. (*n* = 3). *P < 0.05, **P < 0.01, and ***P < 0.001 indicate statistically significant differences from the control group.

We performed TRAP staining to evaluate the effect of SHVB on RANKL-induced osteoclast differentiation. As depicted in Fig. 1a, no cytotoxic effect was observed on bone marrow cells (BMCs) at a test concentration of up to 100 μg/mL SHVB, as determined by the MTT assay (>85% cell survival rate). Consequently, BMCs were allowed to differentiate into osteoclasts in the presence of RANKL and M-CSF for 5 days. Our results demonstrated that SHVB inhibited the formation of TRAP-positive cells during RANKL-induced osteoclast differentiation in a concentration-dependent manner (Fig. 1b).

### SHVB reduced bone turnover markers in serum

**Fig. 2:**
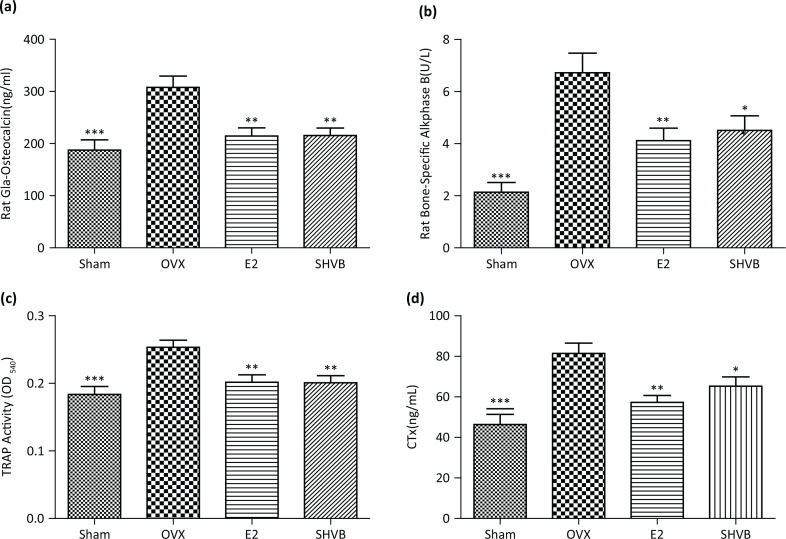
SHVB reduces bone turnover markers in the serum of ovariectomized rats. (a) OCN; (b) BALP; (c) TRAP; (d) CTx. Each value represents the mean ± S.E. for *n* = 10. *P < 0.05, **P < 0.01, and ***P < 0.001 versus OVX group.

We also aimed to determine the effects of SHVB on bone metabolic biomarkers in the serum of blood samples collected from OVX rats. The serum levels of the OCN and BALP bone formation markers were shown to be significantly increased in the OVX group compared with the Sham group. However, treatment with SHVB decreased the elevated serum levels of OCN and BALP in OVX rats (*P* < 0.01; Fig. 2a, b). In addition, the serum levels of TRAP and CTx, which are responsible for enhanced osteoclastogenesis and activation of mature osteoclasts for bone resorption, were demonstrated to be increased in the OVX group; however, treatment with SHVB was shown to significantly reduce TRAP activity (*P* < 0.01) and CTx level (*P* < 0.05; Fig. 2c, d).

### SHVB prevented OVX-induced bone loss

**Fig. 3:**
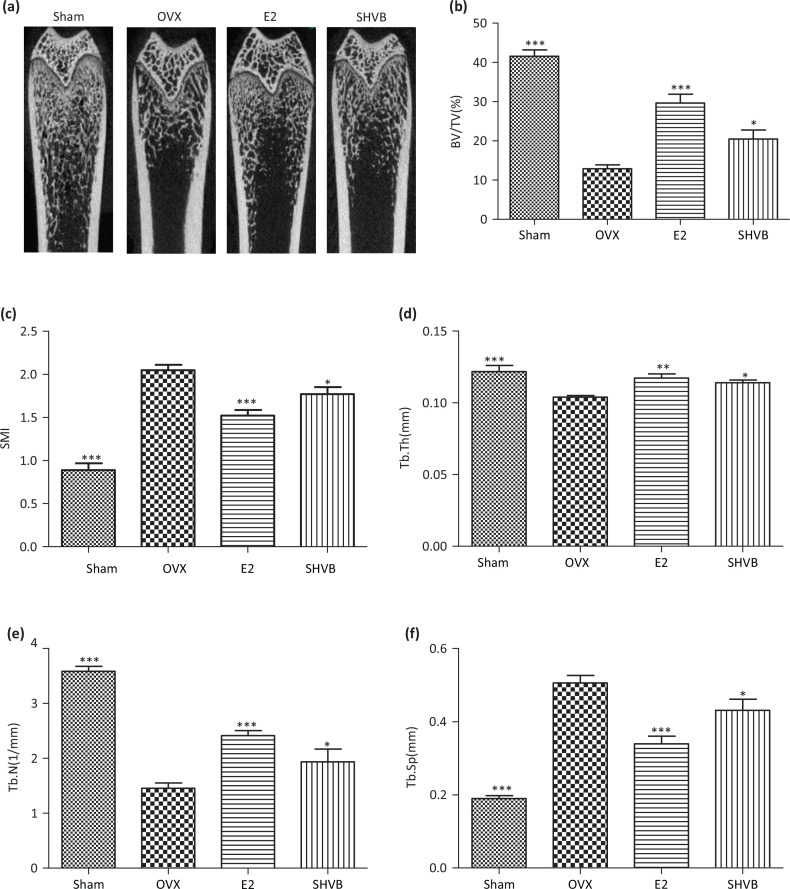
SHVB prevents OVX-induced bone loss. Rats were treated for 12 weeks. (a) Two-dimensional microcomputed tomography (micro-CT) images of the femoral trabecular bone of distal femurs; (b) bone volume/tissue volume (BV/TV); (c) structure model index (SMI); (d) trabecular thickness (Tb.Th); (e) trabecular number (Tb.N); and (f) trabecular separation (Tb.Sp) as analyzed using the micro-CT Skyscan CTAn software. Data are expressed as mean ± S.E. (*n* = 10). *P < 0.05, **P < 0.01, and ***P < 0.001 versus OVX rats.

We primarily evaluated the antiosteoporotic activity of SHVB, performing an *in vivo* experiment employing an OVX-induced bone loss rat model. As illustrated in Fig. 3, OVX caused a significant deterioration of the trabecular bone architecture compared with the Sham group (*P* < 0.001). However, treatment with E2 and SHVB was demonstrated to reduce this OVX-induced alteration. Specifically, treatment with SHVB significantly increased the BV/TV, Tb.Th, and Tb.N compared with the values measured in OVX rats (*P* < 0.05; Fig. 3b, d, e, respectively). In contrast, SMI and Tb.Sp were shown to be higher in the OVX group relative to the Sham group (*P* < 0.001). Treatment with SHVB decreased SMI and Tb.Sp compared with those in the OVX group (*P* < 0.05; Fig. 3c, f, respectively).

### Effect of SHVB on histological morphology of femur bone

**Fig. 4:**
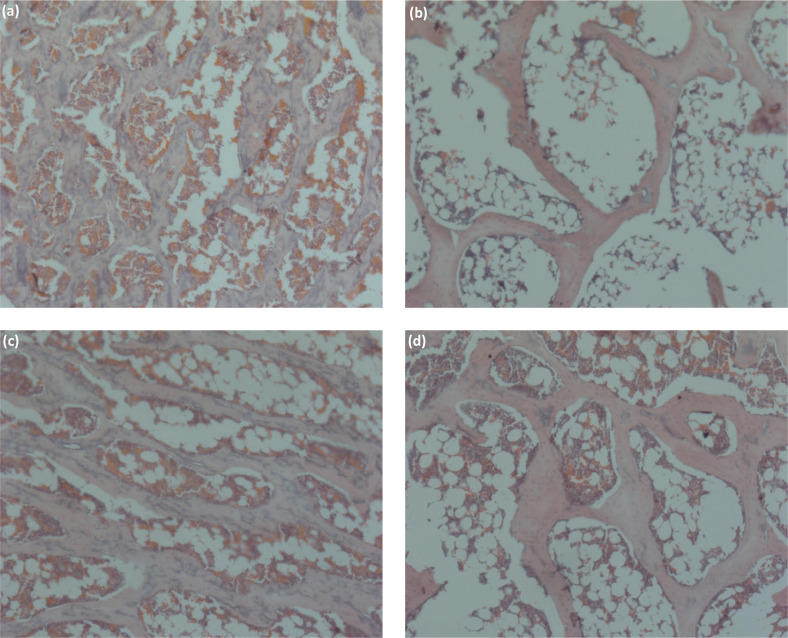
Histology of the trabecular bone region of the femur in rats. (a) Sham; (b) OVX; (c) E2; (d) SHVB. Sections were hematoxylin and eosin (H&E) stained; scale bar = 100 μm. Red arrow indicates trabecular bone.

Bone turnover is a life-long process involving two remodeling processes, namely, bone resorption and formation. In this study, we investigated the histomorphology of the femur bone region of OVX rats using H&E staining. Figure 4 shows a representative histological section of the femur bone region of Sham and OVX rats, as well as those treated with E2 and SHVB. Ovariectomy led to markedly reduced trabecular BV and increased the marrow space (Fig. 4b). Sections from the femur bone region of Sham rats showed the presence of thick trabecular bone with relatively scant marrow space (Fig. 4a). In contrast, the femur region of the OVX group was demonstrated to be small, thin, and sparse compared with those of the Sham group (Fig. 4b). This effect was reversed following a 12-week treatment with E2 and SHVB, both of which resulted in markedly increased trabecular BV (Fig. 4c, d), although E2 was shown to be more potent than SHVB.

### SHVB decreased the tail skin temperature in OVX rats

**Fig. 5:**
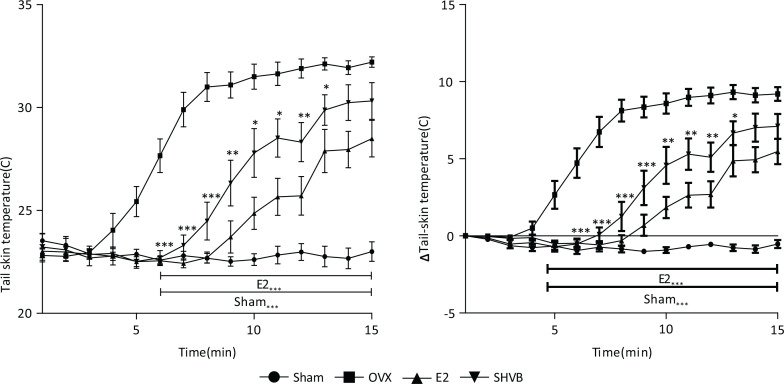
SHVB decreases the tail skin temperature in OVX rats. (a) Tail skin temperature and (b) ΔTail skin temperature. Data are expressed as mean ± S.E. (*n* = 10). *P < 0.05, **P < 0.01, and ***P < 0.001 versus OVX rats.

Results of the measurements of the TST are presented in Fig. 5. The TSTs of OVX rats were observed to start to elevate 3 min after measurement, reaching peak values at 13 min, whereas the TSTs of Sham rats did not show any changes. The TST was maximally elevated at 13–16 min after measurement, with the TSTs of Sham rats being lower than those of the OVX rats. Likewise, SHVB rats were demonstrated to exhibit significantly (*P* < 0.05) lower skin temperatures than those of the OVX group. Treatment with E2 was shown to significantly (*P* < 0.001) inhibit the elevation of the skin temperature in OVX rats.

### SHVB did not affect uterus weight and serum estradiol

**Fig. 6:**
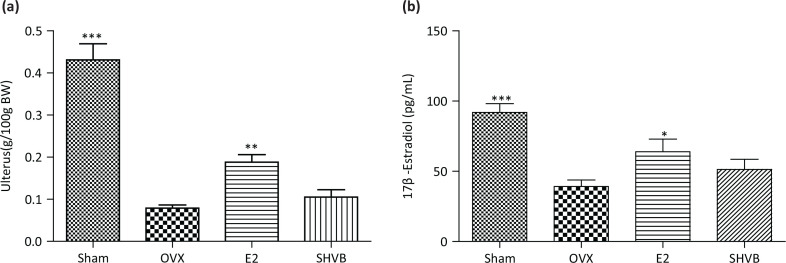
SHVB did not affect uterus weight and serum estradiol. (a) Uterus index and (b) estradiol. Data are expressed as mean ± S.E. (*n* = 10). *P < 0.05, **P < 0.01, and ***P < 0.001 versus OVX rats.

We did not observe any significant weight increase or decrease in liver, spleen, and kidney following the 4 weeks of continuous oral administration (data not shown). As expected, the uterus weight was noted to be significantly (*P* < 0.01) reduced in the OVX group compared with those of the Sham group. Accordingly, administration of E2 was shown to prevent the loss of uterine weight compared with those observed in the OVX group (*P* < 0.01), but still resulted in a lower uterine weight than those of the Sham group, whereas treatment with SHVB did not have any uterotrophic effect (Fig. 6a). The serum concentration of estradiol in OVX and Sham rats is shown in Fig. 6b. The level of estradiol in Sham rats was observed to be significantly higher (*P* < 0.001) than those in OVX rats. This lower level of estradiol was significantly (*P* < 0.05) increased following the administration of E2 but was not affected by treatment with SHVB.

## Standardization of SHVB

**Fig. 7:**
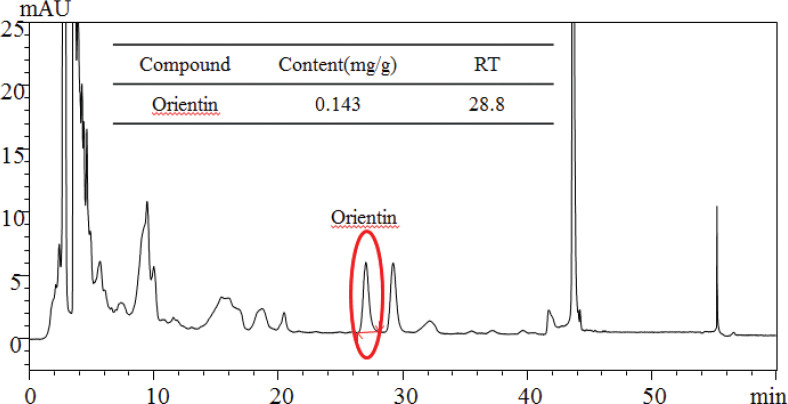
Representative ultra-fast liquid chromatography (UFLC) chromatograms of SHVB. Mobile phase consisted of solvents A (acetonitrile) and B (water) run at a flow rate of 1.0 mL/min. Elution conditions were as follows: 0–40 min, 14–16% A; 40–42 min, 16–100% A; 42–52 min, 100% A; 52–53 min, 100–14% A; 53–60 min, 14% A; and finally, the column was washed and reconditioned. Sample injection volume was 10 μL. Optimum UFLC separation was achieved at 35°C and monitored at 350 nm. AU: arbitrary unit.

As shown in Fig. 7, SHVB was standardized based on the Orientin content, which was determined using high-performance liquid chromatography. The standardized SHVB relative to the main ingredients Orientin (*R* ^2^ = 0.9994) indicated good linearity, while their retention times were typically detected near 28.8 min. The mean Orientin content in the SHVB was approximately 0.143 mg/g of extract.

## Discussion

Climacteric symptoms refer to abnormal conditions related to estrogen deprivation during postmenopausal period, including cognitive impairment, insomnia, depression, irritability, fatigue, psychological symptoms, and increased risk for osteoporosis and cardiovascular disease (42). The climacteric state is associated with an increased risk for metabolic diseases such as heart disease, diabetes, obesity, and hypertension. Hormone therapy is often employed on climacteric disturbance. However, recent report shows that estrogen increases risk of developing phlebothrombosis and coronary artery disease. Moreover, in case of more than 5 years’ treatment, it can cause a high incidence of breast cancer (43). Therefore, non-hormonal or herbal medicine therapy has gained attention as alternative therapies using natural sources. In this study, we investigated the anti-osteoporotic effect of SHVB on RANKL-induced osteoclastogenesis *in vitro* and ovariectomy-induced bone loss *in vivo*. In addition, we demonstrated the anti-hot flush effects of SHVB in an OVX rat model.

Bone remodeling is regulated by a balance between the formation of new bone by osteoblasts and resorption of old bone by osteoclasts. As such, an imbalance in osteoclastogenesis could cause bone loss resulting in osteoporosis (44, 45). Both the M-CSF and RANKL are important cytokines that are known to induce differentiation of osteoclast precursors into activated osteoclasts (46). We demonstrated that SHVB could effectively inhibit RANKL-induced osteoclast differentiation, resulting in anti-osteoporotic effects *in vitro* (Fig. 1). Osteoporosis is a disease characterized by bone loss and the deterioration of bone tissue, with patients exhibiting lower bone density and bone mass than healthy individuals (47, 48). Bone loss triggered by estrogen decline in experimental animals and humans has been reported to result from an increase in osteoclastic bone resorption (49). Hence, OVX rats, which exhibit most of the characteristics of human postmenopausal osteoporosis (50), are widely used as a model to evaluate potential osteoporosis treatments (51).

Quantifiable properties of bone which have been shown to independently predict the occurrence of a future osteoporotic fracture include BMD and bone microarchitecture, that is, trabecular BV, number, and thickness (52). Ovariectomy has been typically linked to deteriorations in trabecular structure and BMD (53). Such a deterioration of trabecular 3D microarchitecture is apparent in the OVX mouse model (54). Therefore, we selected microstructural parameters, such as Tb.Th, Tb.Sp, Tb.N, BV/TV, connectivity density (Conn.D), and SMI (55), to measure the microstructure of the trabecular bone. Our results revealed that SHVB could improve bone quality in the OVX rat model. More specifically, we found that SHVB prevented microstructural parameters deterioration in the distal femur of rats. Oral administration of SHVB inhibited bone loss in OVX rats, indicating that SHVB was effective not only in preserving bone mass but also in rescuing the deterioration of the bone microarchitecture associated with OVX rats (Fig. 3, 4).

The BALP, which is associated with osteoblast activity, has been used to assess bone formation. Likewise, OCN, a critical non-collagenous protein synthesized by osteoblasts, is primarily deposited in the bone extracellular matrix, and therefore it has been considered a specific marker of the function of osteoblasts (56,57).

The crosslinked telopeptides are known to be released from the degradation of type I collagen by proteases during bone resorption, leading to the generation of neoepitopes (CTX-I, ICTP, and NTX-I). Compared with ICTP, the CTX-I and N-terminal telopeptide (NTX-I) have been reported to reflect different enzymatic pathways of bone breakdown, that is, cleavage of collagen type I by either cathepsin K or MMP-1, respectively (11). Based on these, the serum levels of BALP and OCN are used as phenotypic biomarkers of osteoblastic activity, whereas CTx and TRAP are widely accepted biomarkers of bone resorption. Serum concentrations of BALP, OCN, CTx, and TRAP in OVX rats were observed to be significantly higher than those in the Sham group, whereas the 12-week treatment with E2 or SHVB lowered the increased bone turnover (Fig. 2), which was evidenced by the significant decreases noted in the levels of BALP, OCN, CTx, and TRAP. In this study, we found that SHVB prevented bone loss, and assumed that this effect was manifested through the decrease in bone turnover. Finally, we showed that SHVB inhibited osteoclast differentiation, prevented bone mass reduction, and improved the trabecular bone structure and biochemical markers in OVX-induced osteoporosis.

Hot flush symptoms can affect women’s work and social life, sleep pattern, and overall health (58). Flushing of the tail skin in OVX animals is regarded as a good indicator for climacteric hot flushes, although the spontaneous appearance of flushing is irregular. The TST has been shown to increase following ovariectomy, with the effect being reduced by supplementation of estrogen (59–62).

In this study, SHVB inhibited the elevation of TST in OVX rats (Fig. 5) but did not restore the lowered estrogen serum levels. Moreover, uterine hypertrophy did not occur (Fig. 6). These findings indicate that SHVB did not confer estrogenic activity to serum, and it did not potentiate estrogen-production in the extra ovular tissue in OVX rats.

Therefore, it is expected that SHVB will be promising as a novel alternative treatment for relieving climacterium symptoms, especially hot flush and osteoporosis in menopausal women. The screening of biologically active compounds should be conducted in future with focus on the detailed mechanism.

## Conclusions

Hot flushes and osteoporosis are primary symptoms of menopause. In this study, we have shown that SHVB administration to osteoporotic rats suppressed deteriorations of the trabecular bone microstructure and improved bone turnover biochemical markers, such as BALP, OCN, TRAP, and CTx. Furthermore, SHVB treatment alleviated the increase in the TST of the estrogen-deficient animals. Taken together, this study demonstrated the beneficial effect of SHVB in improving menopause, and that SHVB has a potential of being a natural hormone replacement substance.
